# First Report on the Chemical Composition and Biological Activities of the Leaf Essential Oil of *Glycosmis petelotii* From Vietnam

**DOI:** 10.1155/sci5/4082933

**Published:** 2026-11-15

**Authors:** Ton That Huu Dat, The-Huan Tran, Linh Thuy Thi Tran, Vu Thi Thanh Tam, Tran Phuong Ha, Diem Tran Phan Thi, Le Tuan Anh, Ty Viet Pham, Le Canh Viet Cuong

**Affiliations:** ^1^ Institute of Advanced Technology in Central Vietnam Institute of Advanced Technology Vietnam Academy of Science and Technology 321 Huynh Thuc Khang Hue Vietnam vast.ac.vn; ^2^ Faculty of Pharmacy Hue University of Medicine and Pharmacy, Hue University 06 Ngo Quyen Hue Vietnam; ^3^ Faculty of Chemistry University of Education Hue University 34 Le Loi Hue Vietnam hueuni.edu.vn

**Keywords:** *α*-glucosidase inhibition, antibacterial activity, essential oil, *Glycosmis petelotii*

## Abstract

This study presents the first report on the chemical composition and biological activities of the essential oil (EO) extracted from the leaves of *Glycosmis petelotii*. Gas chromatography–mass spectrometry (GC–MS) analysis of leaf EO of *Glycosmis petelotii* revealed that monoterpene hydrocarbons were predominant constituents (59.23%), followed by sesquiterpene hydrocarbons (18.22%), oxygenated sesquiterpenes (5.25%), and oxygenated monoterpenes (1.10%). The major components identified were limonene (55.56%), tentatively identified as niranin (15.68%), *β*‐caryophyllene (8.70%), and bicyclogermacrene (5.03%). Antibacterial assays revealed that the EO exhibited antibacterial activity against *Escherichia coli*, *Salmonella enterica*, *Pseudomonas aeruginosa*, *Enterococcus faecalis*, *Staphylococcus aureus*, and *Bacillus cereus*, with MIC values ranging from 128 to 256 μg/mL. Subsequent bioassays demonstrated *α*‐glucosidase inhibitory activity of the EO, with an IC_50_ value of 707.5 ± 22.6 μg/mL, compared to the standard inhibitor acarbose (IC_50_ = 117.7 ± 4.0 μg/mL). However, the EO exhibited weak inhibitory activity against acetylcholinesterase, xanthine oxidase, and nitric oxide production, with IC_50_ values > 100 μg/mL. Molecular docking indicated that the four selected major constituents could adopt plausible binding poses within the *α*‐glucosidase active site through different noncovalent interactions. Their predicted binding affinities ranged narrowly from −6.1 to −6.4 kcal/mol, indicating broadly comparable computational values among the compounds.

## 1. Introduction

The genus *Glycosmis* (Rutaceae) is primarily distributed in tropical and subtropical regions of Asia to northwestern Australia, with approximately 55 species recorded [1]. Many *Glycosmis* species have long been utilized in traditional medicine. Notably, *G. pentaphylla* has been traditionally prescribed for the treatment of rheumatism, fever, dermatological disorders, hepatic ailments, and inflammatory conditions [2], whereas *G. arborea* has been used in the treatment of helminthiasis, eczema, fever, rheumatism, wounds, cough, bronchitis, and various skin diseases [3, 4].

Phytochemical investigations of the genus have led to the isolation of diverse secondary metabolites, including alkaloids, sulfur‐containing amides, terpenoids, flavonoids, phenolics, anthranilamides, cinnamamides, and quinones [5]. Furthermore, pharmacological investigations of *Glycosmis* extracts and isolated compounds have demonstrated a wide array of biological activities, such as antioxidant [6], anti‐inflammatory [7–9], anti‐Alzheimer’s [10], cytotoxic [6, 11–13], antimicrobial [6, 13, 14], antidiabetic [13], hepatoprotective [15], and antiviral activities [16, 17].

*G. petelotii* is a plant native to Vietnam, typically found in humid tropical forests. In traditional Vietnamese medicine, *G*. *petelotii* is used to treat a variety of ailments, including cough, rheumatism, fever, eczema, skin diseases, and snake bites [18]. Despite its widespread use in folk Vietnamese medicine, investigations into the chemical composition and biological activities of *G. petelotii* remain limited. Recent phytochemical investigations of this species have led to the isolation of sulfur‐containing alkaloids that inhibit phenylephrine‐induced contraction [19, 20]. Therefore, further investigation into the chemical composition of the essential oil (EO) from *G. petelotii* is needed to identify bioactive constituents, explore its potential pharmacological properties, and support the development of applications in medicine and the food industry. This study aimed to characterize the chemical composition of *G*. *petelotii* leaf EO, evaluate its biological activities, and explore the potential interactions of its major constituents with the molecular target associated with the most promising inhibitory activity through molecular docking. To the best of our knowledge, this is the first investigation into both the chemical profile and biological properties of the EO derived from *G. petelotii* leaves.

## 2. Material and Methods

### 2.1. Plant Materials

Leaves of *G. petelotii* (Figure 1) were collected in June 2025 from Huong Hoa District, Quang Tri Province (16°47′13″N, 106°35′09″E). The species was identified by Dr. Le Tuan Anh from the Institute of Advanced Technology in Central Vietnam. A voucher specimen (MISR‐NCXS‐27) has been deposited at the Institute of Advanced Technology in Central Vietnam.

**FIGURE 1:**
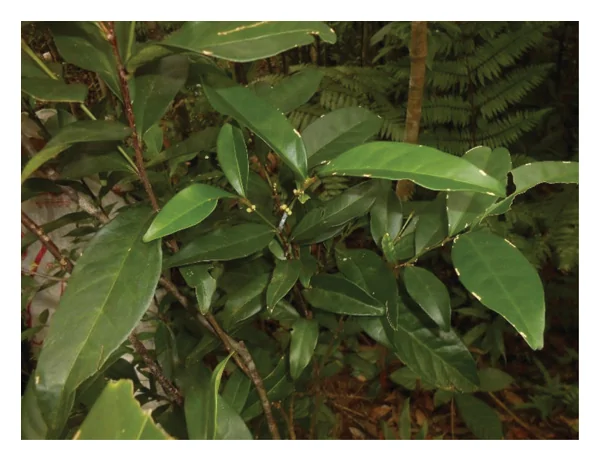
*Glycosmis petelotii* in its habitat.

### 2.2. Chemicals

Lipopolysaccharides (LPS) from *Escherichia coli*, sodium nitrite, sulfanilamide, N‐1‐naphthylethylenediamine dihydrochloride, dimethyl sulfoxide (DMSO), *α*‐glucosidase, xanthine oxidase (XO), acetylcholinesterase (AChE), *p*‐nitrophenyl‐α‐D‐glucopyranoside (pNPG), acarbose, xanthine, allopurinol, acetylthiocholine iodide, 5,5‐dithiobis‐2‐nitrobenzoate (DTNB), galantamine, and dexamethasone were purchased from Sigma Chemical Co. (St. Louis, MO, USA). Dulbecco’s Modified Eagle’s Medium (DMEM) and fetal bovine serum (FBS) were from Life Technologies, Inc. (Gaithersburg, MD, USA). The Griess Reagent System was supplied by Promega Corporation (WI, USA).

### 2.3. Hydrodistillation of *G. petelotii* EO

Hydrodistillation was carried out with minor modifications according to a previously reported method [21].

Fresh *G. petelotii* leaves collected from a single sampling batch were thoroughly mixed and divided into three 300‐g portions for independent hydrodistillation experiments. Each portion was mixed with 3000 mL of distilled water (plant material‐to‐water ratio, 1:10, w/v) in a 5‐L flask and hydrodistilled for 3 h using a Clevenger apparatus equipped with a 10‐mL graduated oil receiver. The volume of EO obtained from each distillation was read directly from the graduated receiver, and the oil yield was expressed as milliliters per 100 g of fresh leaves. The reported oil yield represents the mean ± standard deviation (SD) of three independent hydrodistillations. The EOs obtained from the three independent distillations were pooled, dried over Na_2_SO_4_, transferred into a tightly sealed dark glass vial with minimal headspace, protected from light, and stored at 4°C. The interval between distillation, GC–MS analysis, and biological assays was approximately 10 days.

### 2.4. GC–MS Analysis

The *G. petelotii* EO composition was analyzed using a GC/MS‐QP2010 Plus system (Shimadzu), which was equipped with an Equity‐5 capillary column and coupled to a QP2010 Plus mass spectrometer [21].

The oil was diluted 100‐fold with *n*‐hexane. A 1‐μL aliquot of the diluted sample was injected in splitless mode. The analysis used helium as the carrier gas at a flow rate of 1.5 mL/min. A temperature of 280°C was applied to both the injector and interface. The analysis started with the column temperature at 60°C for 2 min. It was then increased to 240°C at 3°C/min and held for 10 min. Finally, the temperature was raised to 280°C at 5°C/min and maintained for 40 min. The ionization voltage was set at 70 eV. The mass range was scanned from 45 to 500 m/z. The sampling rate was set at 1.0 scan s^−1^. The volatile constituents were tentatively identified through comparison of their mass spectra with published literature data [22] and with mass spectral libraries (NIST 2018 and WILEY 7). A homologous series of *n*‐alkanes (*C*_8_–*C*_38_) was analyzed under identical conditions and used as the reference for calculating the experimental retention indices (Figure S3). The quantities were estimated from the relative percentage of peak areas. The percentages of volatile compound groups were calculated by summing the relative percentages of identified compounds and classifying them into each group.

### 2.5. Biological Activity Assays

The biological activities of the EO were evaluated following the protocols detailed in the supporting information, including assays for antimicrobial activity [23], *α*‐glucosidase inhibition [21], XO inhibition [24], AChE inhibition [25], and nitric oxide production inhibition [26]. The principal experimental conditions are summarized below, with detailed procedures and control settings provided in Supporting Information 1–5.

#### 2.5.1. Antibacterial Activity

Antibacterial activity was determined against six reference strains, including *Salmonella enterica* ATCC 13076, *Escherichia coli* ATCC 25922, *Pseudomonas aeruginosa* ATCC 27853, *Enterococcus faecalis* ATCC 29212, *Bacillus cereus* ATCC 14579, and *Staphylococcus aureus* ATCC 25923. MICs were determined by broth microdilution in Mueller–Hinton broth with an EO concentration range of 0.5–512 μg/mL. The final bacterial inoculum was 5 × 10^5^ CFU/mL. Plates were incubated at 37°C for 24 h, and bacterial growth was monitored at 630 nm. Ciprofloxacin was used as the reference antibiotic. Appropriate growth, sterility, solvent, and sample blank controls were included in Supporting Information 1.

#### 2.5.2. Enzyme Inhibition Assays

α‐Glucosidase, XO, and AChE inhibition were examined using microplate spectrophotometric methods. For *α*‐glucosidase, EO was reacted with *α*‐glucosidase (0.5 U/mL) in 0.1 M phosphate buffer (pH 6.8) at 37°C, followed by the addition of pNPG (5 mM). The reaction was then terminated by the addition of 50 μL of 0.2 M Na_2_CO_3_. Absorbance was measured at 405 nm. Acarbose served as the positive control. XO inhibition was assessed using bovine milk XO (0.01 U/mL) in 70 mM phosphate buffer (pH 7.5), with xanthine (150 μM) as the substrate. After incubation at 25°C, the reaction was terminated with 1 N HCl and read at 290 nm. Allopurinol was used as the reference inhibitor. AChE activity was measured using electric eel AChE (0.2 U/mL), with DTNB and acetylthiocholine iodide as the reaction components. After incubation at 25°C, the reaction was terminated by the addition of 20 μL of 4% SDS. Absorbance was monitored at 415 nm, with galantamine as the positive control. Inhibition was calculated relative to the corresponding untreated controls. Detailed reaction compositions and calculation procedures are given in Supporting Information 2–4.

#### 2.5.3. Inhibition of Nitric Oxide Production

The anti‐inflammatory effect of the EO was evaluated using LPS‐stimulated RAW264.7 macrophages and the Griess assay. Cells (1 × 10^5^ cells/well) were pretreated with the EO for 1 h and subsequently stimulated with LPS (1 μg/mL) for 24 h. Nitrite accumulation in the culture medium was quantified at 540 nm using a NaNO_2_ standard curve. Dexamethasone was used as the positive control. Cell viability was assessed in parallel by the MTT assay to exclude effects associated with reduced cell viability. Detailed and calculation procedures are given in Supporting Information 5.

All biological assays were performed in three independent experiments (*n* = 3), and the results are expressed as mean ± SD. IC_50_ values were determined by nonlinear regression analysis using a four‐parameter logistic (4PL) dose–response model in GraphPad Prism 8.0. The IC_50_ values were calculated from the fitted concentration–response curves.

### 2.6. Molecular Docking Methodology

Molecular docking was employed as an exploratory computational approach to examine the predicted binding poses and interaction profiles of selected major constituents of *G. petelotii* EO within the *α*‐glucosidase binding site. The docking analysis was designed as a focused evaluation of constituents present at ≥ 5% of the total EO composition.

Molecular docking simulations were performed using AutoDock Vina 1.1.2 [27]. The crystal structure of *α*‐glucosidase from *Bacillus* was retrieved from the Protein Data Bank (PDB ID: 5ZCC) [28]. This structure was selected to match the biological source of the *α*‐glucosidase used in the in vitro inhibition assay. The protein was preprocessed using AutoDock Tools 1.5.7 [29]. Water molecules were deleted, polar hydrogens were introduced, and Kollman charges were assigned. The cocrystallized ligand was removed from the binding site and retained separately for validation of the docking protocol. The structure was subsequently saved in the *.pdbqt* format. The structures of the ligands were drawn using ChemDraw 16.0 and converted to 3D structures. Geometry optimization was performed using Open Babel 3.1.1 with the MMFF94 force field, and the final ligand files were prepared in *.pdbqt* format for docking.

Docking simulations were conducted targeting the active site of the enzyme, with the grid box centered at (*x* = 4.08, *y* = 50.719, *z* = 74.087) and dimensions set to 20.0 × 20.0 × 20.0 Å along the *x*‐, y‐, and *z*‐axes. All other parameters were maintained at their default values.

The docking protocol was assessed by redocking the cocrystallized maltose into the original *α*‐glucosidase active site under the same docking conditions. The resulting pose was aligned with the crystallographic ligand, and the heavy‐atom RMSD was determined using Open Babel.

The docking results were evaluated based on the predicted binding affinity (ΔG, kcal/mol), and interaction profiles were analyzed using UCSF ChimeraX 1.9 [30] and Discovery Studio Visualizer 2024.1.

## 3. Results and Discussion

### 3.1. Chemical Composition of *G. petelotii EO*

The average yield of EO from *G. petelotii* leaves was 0.12 ± 0.02% fresh weight (v/w). This EO is lighter than water, is pale yellow in color, and possesses the characteristic aroma of the *Glycosmis* species. Twenty‐three volatile compounds were identified by comparing GC–MS spectra with previously published data and the WILEY 7 and NIST 2018 spectral libraries, representing 99.48% of the EO content (Table 1 and Figure 2).

**TABLE 1:** Volatile compounds identified in the EO of *G. petelotii* leaves.

No	RT	Compounds	RI_E_	RI_L_	Content (%)	Classification
1	7.14	*α*‐Pinene	931	932	0.40	MH
2	9.08	Myrcene	989	988	0.81	MH
**3**	**10.58**	**Limonene**	**1027**	**1024**	**55.56**	MH
4	10.92	*cis*‐Ocimene	1035	1032	1.45	MH
5	11.35	*trans*‐Ocimene	1046	1044	1.01	MH
6	13.57	Linalool	1099	1095	0.81	OM
7	14.29	*endo*‐Fenchol	1115	1114	0.29	OM
8	25.83	*α*‐Copaene	1377	1374	0.36	SH
9	26.43	*β*‐Cubebene	1391	1387	0.27	SH
10	26.52	*β*‐Elemene	1393	1389	0.65	SH
**11**	**27.69**	***β*-Caryophyllene**	**1421**	**1419**	**8.70**	SH
12	29.11	α‐Humulene	1455	1454	1.20	SH
13	30.24	Germacrene D	1483	1480	0.18	SH
**14**	**30.89**	**Bicyclogermacrene**	**1498**	**1500**	**5.03**	SH
15	31.34	*β*‐Bisabolene	1510	1505	1.35	SH
16	31.95	*δ*‐Cadinene	1525	1523	0.48	SH
17	34.09	Spathulenol	1580	1577	1.53	OS
18	34.32	Caryophyllene oxide	1586	1582	1.30	OS
19	34.66	Viridiflorol	1594	1592	0.37	OS
20	35.10	Khusimone	1606	1604	0.38	OS
21	36.35	3‐*iso*‐Thujopsanone	1639	1641	0.23	OS
22	36.51	Cedrelanol	1644	1640	1.44	OS
**23**	**39.49**	Niranin**^∗^**	**1725**	**1715**	**15.68**	**NT**
Total					99.48	
Monoterpene hydrocarbon (MH)					59.23	
Oxygenated monoterpene (OM)					1.10	
Sesquiterpene hydrocarbon (SH)					18.22	
Oxygenated sesquiterpene (OS)					5.25	
Nonterpenic compounds (NT)					15.68	

*Note:* RI_E_: experimental retention indices, RI_L_: literature retention indices; mass spectrum (MS) was based on electron ionization (EI) mode. Bold indicates major compounds (≥ 5%).

Abbreviation: RT = retention time.

^∗^Tentatively identified based on comparison of the EI mass spectrum and retention index (Figures S1–S2 and Table S3).

**FIGURE 2:**
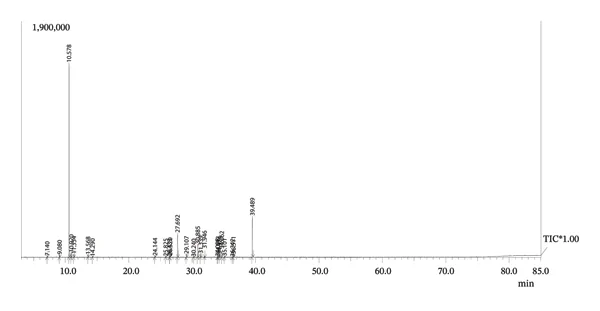
The GC chromatogram of *G. petelotii* EO.

Monoterpene hydrocarbons (59.23%), along with sesquiterpene hydrocarbons (18.22%), oxygenated sesquiterpenes (5.25%), and oxygenated monoterpenes (1.10%), are the main constituents of *G. petelotii* leaf EO. The major compounds identified included limonene (55.56%), tentatively identified as niranin (15.68%), *β*‐caryophyllene (8.70%), and bicyclogermacrene (5.03%). In addition, notable constituents with significant amounts included spathulenol (1.53%), *cis*‐ocimene (1.45%), cedrelanol (1.44%), and *β*‐bisabolene (1.35%).

Substantial differences in the presence of major compound groups and characteristic constituents have been revealed in previous studies analyzing the chemical composition of EOs from many species of the genus *Glycosmis*. The EO of the leaves of *G. puberula* var. *eberhardtii* has been reported to contain a high content of sesquiterpene hydrocarbons (63.6%). The remaining compounds, including monoterpene and oxygenated monoterpene hydrocarbons, account for 15.8% and 15.1%, respectively. Among these, *α*‐copaene, *α*‐humulene, (*E*)‐caryophyllene, (*Z*)‐*β*‐ocimene, and linalool are the main compounds in the chemical composition [31]. Meanwhile, the EO from *G. petelotii* mainly contained monoterpene hydrocarbons, with limonene as the dominant compound (55.56%). Moreover, *α*‐humulene, linalool, and *α*‐copaene were present at low concentrations of 1.20%, 0.81%, and 0.36%, respectively. Similarly, the EO obtained from the aerial part of *G. parviflora* exhibited a predominance of sesquiterpenoids (53.0%) and monoterpenoids (26.5%), with (*Z*)‐*β*‐ocimene (8.9%), methyl isoeugenol (11.1%), and (*Z*)‐caryophyllene (20.6%) as the main components [32]. The *G. lucida* EO was notably rich in elixene (19.81%), anethole (12.05%), spathulenol (10.68%), verbenone (10.32%), and *β*‐caryophyllene (6.87%) [33]. These variations underscore the significant chemical diversity present in EOs among species within the *Glycosmis* genus.

According to published studies, niranin has been isolated from *G. mauritiana* leaves; however, its presence has not been reported in the EOs of *Glycosmis* species [34]. In this study, a compound tentatively identified as niranin was detected in the leaf EO of *G. petelotii* at a relatively high abundance (15.68%). If confirmed by further studies, this finding may expand the known chemical composition of the *Glycosmis* genus and highlight the characteristic chemical diversity among its species.

### 3.2. Biological Activities of the EO

Antibacterial assays showed that *G. petelotii* EO inhibited all tested bacterial strains, including *E*. *coli, S*. *enterica, P*. *aeruginosa, E*. *faecalis, S*. *aureus,* and *B*. *cereus*, with MICs in the range of 128–256 μg/mL (Table S1). The major constituents of this oil, such as limonene, *β*‐caryophyllene, and bicyclogermacrene, have each been previously reported to possess antimicrobial activity [35–39] and likely contribute substantially to the oil’s observed antibacterial activity.

The EO from the *G. petelotii* exhibited *α*‐glucosidase inhibitory activity, with an IC_50_ value of 707.5 ± 22.6 μg/mL, compared with acarbose (IC_50_ = 117.7 ± 4.0 μg/mL) (Table S2). The observed *α*‐glucosidase inhibitory activity of the EO may be partly associated with its major constituents, such as limonene (55.56%) and *β*‐caryophyllene (8.70%), which have been reported to exhibit antidiabetic effects by in vitro or in vivo experiments [40–42]. In vitro assays demonstrated that limonene exhibited *α*‐glucosidase inhibitory activity, with an IC_50_ value of 78.03 ± 2.31 μg/mL [40]. Furthermore, limonene has been demonstrated to have uncompetitive inhibition with *α*‐glucosidase [42]. While in vitro evidence that *β*‐caryophyllene inhibits *α*‐glucosidase is lacking, in vivo models consistently demonstrate its antidiabetic potential. Recent in vivo experiments have shown that *β*‐caryophyllene reduced fasting glucose, increased plasma insulin, normalized glycogen content, and restored aberrant carbohydrate metabolic enzymes in the liver, muscle, and kidney in streptozotocin‐induced diabetic rats [43]. Nevertheless, this association remains hypothetical, as the present study did not evaluate the *α*‐glucosidase inhibitory activity of the individual constituents. The observed activity may instead reflect the combined effects, including possible additive or synergistic interactions, of multiple compounds present in the EO.

Further in vitro bioassays showed that *G. petelotii* EO exhibited only weak inhibitory activity against AChE, XO, and nitric oxide production, with IC_50_ values exceeding the highest tested concentration (100 μg/mL) (Table S2). Cell viability remained above 90% at the highest concentration tested (100 μg/mL), indicating that the lack of nitric oxide inhibition was not attributable to cytotoxicity. However, this does not necessarily indicate the absence of bioactive constituents, as bioactive compounds may be present only in small amounts, or the dominance of weakly active compounds could mask the overall bioactivity of the EO. For instance, several components detected in *G. petelotii* EO have been reported to show notable in vitro activities, such as limonene (XO inhibition) [44], *β*‐caryophyllene, *α*‐pinene, and caryophyllene oxide (AChE inhibition) [45–47], as well as *α*‐pinene and linalool (nitric oxide production inhibition) [48–50]. Therefore, studies are warranted to identify promising bioactive compounds in *G. petelotii* EO.

### 3.3. Molecular Docking Results for Major *G. petelotii* Constituents

Among the biological targets evaluated in vitro, *α*‐glucosidase was selected for molecular docking because the EO showed measurable inhibitory activity against this enzyme, with an IC_50_ value of 707.5 ± 22.6 μg/mL, whereas only weak inhibitory activity was observed against AChE, XO, and nitric oxide production. Accordingly, *α*‐glucosidase was considered the most relevant target for exploratory docking because it was the only tested enzyme for which the EO produced a measurable inhibitory response under the experimental conditions. Molecular docking was therefore applied as a complementary computational approach to examine the possible binding modes and interaction profiles of selected major constituents within the *α*‐glucosidase binding site.

The docking protocol was validated by redocking the cocrystallized ligand into its original binding site. The redocked pose reproduced the crystallographic orientation with a heavy‐atom RMSD of 0.91 Å, which was below the predefined acceptance threshold of 2.0 Å and supported the suitability of the protocol for binding‐pose analysis. The superimposed poses are shown in Figure S4.

The analysis included limonene, *β*‐caryophyllene, bicyclogermacrene, and niranin, each present at ≥ 5% of the EO. Together, these four constituents accounted for 84.97% of the identified oil composition and represented its predominant fraction. Because the compound assigned as niranin was only tentatively identified based on GC–MS and retention‐index data, its docking analysis should be regarded as provisional and conditional on the correctness of this structural assignment.

The predicted binding affinities ranged narrowly from −6.1 to −6.4 kcal/mol, indicating broadly comparable values among the four compounds (Table 2). Given the small differences between these values, the compounds were not ranked according to predicted binding affinity.

**TABLE 2:** Predicted binding affinities (Δ*G*) and interaction profiles of selected major constituents of *G. petelotii* EO with *α*‐glucosidase.

Compound	Δ*G* (kcal/mol)	Hydrogen bonds	Hydrophobic interactions	Other interactions
Limonene	−6.1		Tyr63, His103, Phe163, Ala200, Phe282	
*β-*Caryophyllene	−6.4		Ile143, Phe163, Ala200,	
Bicyclogermacrene	−6.4		Ile143, Phe225	
Niranin	−6.1	His203, Asn258	Ala200	Asp199, Phe225

*Note:* Niranin exhibited the most diverse predicted interaction profile among the four compounds. It formed hydrogen bonds with His203 and Asn258, together with pi–sulfur, pi–anion, and pi–alkyl interactions involving Phe225, Asp199, and Ala200, respectively (Figure 3D). These contacts contributed to a plausible predicted orientation of niranin within the binding site.

**FIGURE 3:**
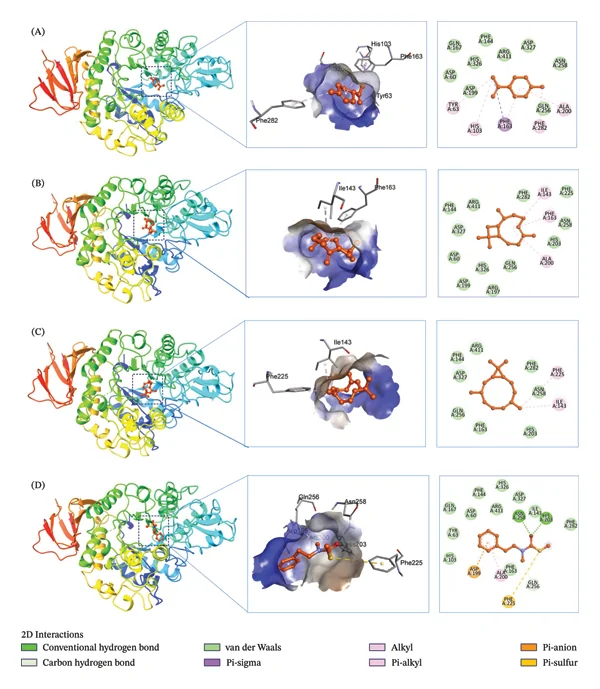
3D and 2D interaction diagrams of *α*‐glucosidase with the major *G. petelotii* EO constituents: (A) limonene, (B) *β*‐caryophyllene, (C) bicyclogermacrene, and (D) niranin.

Limonene interacted predominantly through hydrophobic contacts with Tyr63, His103, Phe163, Ala200, and Phe282 (Figure 3A). These contacts may contribute to stabilization of its predicted binding pose within the enzyme binding site.

*β*‐Caryophyllene showed a predicted binding affinity of −6.4 kcal/mol and interacted mainly through hydrophobic and *van der Waals* contacts involving Ile143, Phe163, and Ala200 (Figure 3B). These contacts characterized its predicted interaction pattern within the binding site.

Bicyclogermacrene also showed a predicted binding affinity of −6.4 kcal/mol and formed mainly hydrophobic contacts with Ile143 and Phe225 (Figure 3C). Although its predicted interaction network was less extensive than that of niranin, its predicted binding affinity was comparable to those of the other investigated compounds.

*Van der Waals* interactions were also observed across the predicted complexes and may contribute to stabilization of the calculated binding poses. Overall, the four selected constituents displayed distinct interaction profiles while yielding narrowly distributed predicted binding affinities. These results provide exploratory information on their possible interactions with *α*‐glucosidase but do not establish their relative inhibitory contributions. Although the four investigated constituents represented 84.97% of the identified oil composition, possible contributions from minor constituents cannot be excluded.

## 4. Conclusion

The EO of *G. petelotii* leaves was characterized by limonene (55.56%), tentatively identified as niranin (15.68%), *β*‐caryophyllene (8.70%), and bicyclogermacrene (5.03%) as its major constituents. The EO exhibited antibacterial activity, with MIC values ranging from 128 to 256 μg/mL, and *α*‐glucosidase inhibitory activity, with IC_50_ values of 707.5 ± 22.6 μg/mL, while only weak inhibition was observed against XO, AChE, and nitric oxide production, with IC_50_ values > 100 μg/mL. Molecular docking indicated that the selected major constituents could adopt plausible binding poses within the *α*‐glucosidase binding site through different noncovalent interactions. These findings provide valuable insights into the chemical profile and biological activities of *G. petelotii* EO.

## Author Contributions

Conceptualization: Le Canh Viet Cuong and Ton That Huu Dat; methodology: The‐Huan Tran, Le Canh Viet Cuong, and Ton That Huu Dat; supervision: Le Canh Viet Cuong and Ton That Huu Dat; sampling, extraction, and biological testing: The‐Huan Tran, Vu Thi Thanh Tam, Phan Thi Diem Tran, Phan Thi Diem Tran, Ty Viet Pham, Linh Thuy Thi Tran, and Le Tuan Anh; plant identification: Le Tuan Anh; molecular modeling: The‐Huan Tran and Linh Thuy Thi Tran; data curation: The‐Huan Tran, Le Canh Viet Cuong, and Ton That Huu Dat; original draft preparation: Linh Thuy Thi Tran, The‐Huan Tran, Ty Viet Pham, Le Canh Viet Cuong, and Ton That Huu Dat; writing–review and editing: The‐Huan Tran, Le Canh Viet Cuong, and Ton That Huu Dat.

## Funding

This research is funded by Vietnam Academy of Science and Technology (VAST), under the grant number NCXS02.04/25–26.

## Conflicts of Interest

The authors declare no conflicts of interest.

## Supporting Information

Additional supporting information can be found online in the Supporting Information section.

## Supporting information


**Supporting Information** Supporting Table S1: Antibacterial activity of the *Glycosmis petelotii* essential oil. Supporting Table S2: Biological activity of the *Glycosmis petelotii* essential oil. Supporting Table S3: Comparison of the experimental and reference EI mass spectra of niranin. Supporting Figure S1: Experimental EI mass spectrum of the peak tentatively identified as niranin. Supporting Figure S2: Experimental EI mass spectrum of niranin from Adams (2017). Supporting Figure S3: The TIC chromatogram of the n‐alkane standard mixture. Supporting Figure S4: Superimposition of the crystallographic and redocked poses of maltose within the *α*‐glucosidase active site. The crystallographic pose is shown in blue, and the redocked pose is shown in yellow. The heavy‐atom RMSD between the two poses was 0.91 Å.

## Data Availability

The datasets used and/or analyzed during the current study are available from the corresponding author upon reasonable request.
