# Supplementary material for: First Report on the Chemical Composition and Biological Activities of the Leaf Essential Oil of Glycosmis petelotii From Vietnam
Source: Scientifica (Cairo). 2026 Nov 15;2026:4082933. doi: 10.1155/sci5/4082933 (PMC13770276; doi:10.1155/sci5/4082933)
Supplement: Supplementary file 1 [file SCI5-2026-4082933-s001.docx]

**Supplementary Information**

**First report on the chemical composition and biological activities of the leaf essential oil of *Glycosmis petelotii* from Vietnam**

Ton That Huu Dat^1,#^, The-Huan Tran^2,#^, Linh Thuy Thi Tran^2^, Vu Thi Thanh Tam^1^, Tran Phuong Ha^1^, Phan Thi Diem Tran^1^, Le Tuan Anh^1^, Ty Viet Pham^3^, Le Canh Viet Cuong^1,*^

*^1^Institute of Advanced Technology in Central Vietnam, Institute of Advanced Technology, Vietnam Academy of Science and Technology, 321 Huynh Thuc Khang, Hue City, Vietnam*

*^2^Faculty of Pharmacy, Hue University of Medicine and Pharmacy, Hue University, 06 Ngo Quyen, Hue City, Vietnam*

*^3^Faculty of Chemistry, University of Education, Hue University, 34 Le Loi, Hue City, Vietnam*

Correspondence should be addressed to Le Canh Viet Cuong; e-mail: lcvcuong@iat.vast.vn

**Antibacterial assay**

Antibacterial activity of the essential oil was evaluated against six bacterial strains, including Gram-negative bacteria, *Salmonella enterica* ATCC13076, *Escherichia coli* ATCC25922, *Pseudomonas aeruginosa* ATCC27853, and Gram-positive bacteria *Enterococcus faecalis* ATCC29212, *Bacillus cereus* ATCC14579 and *Staphylococcus aureus* ATCC25923. Minimal inhibitory concentrations (MIC) of the essential oil against the tested bacterial strains were determined using broth microdilution in 96-well plates ^[1]^. Briefly, samples were dissolved in dimethyl sulfoxide (DMSO) and subsequently subjected to serial two-fold dilution within Mueller-Hinton (MH) broth to a concentration range that was twice the final concentration. Equal volumes of the diluted sample and bacterial suspension were mixed in each well, resulting in final essential-oil concentrations of 0.5–512 µg/mL and a final bacterial inoculum of 5 × 10^5^ CFU/mL. The final DMSO concentration in all assay wells was less than 0.5% (v/v).

A solvent control consisting of MH broth, bacterial suspension, and DMSO at the same final concentration as the test wells was included to confirm that the solvent did not affect bacterial growth. A growth control containing MH broth and bacterial suspension without essential oil and a sterility control containing MH broth only were included in every experiment. Essential-oil blank wells containing MH broth, essential oil, and DMSO but no bacteria were also included to correct for background absorbance.

The plates were incubated at 37 ^o^C for 24 h, and then the absorbance at 630 nm was measured using a microplate reader (BioTek Instruments, Winooski, VT, USA). The lowest concentration of the sample which prevents the growth of the bacteria was defined as the minimal inhibitory concentrations (MIC). Ciprofloxacin was used as a positive control. MIC values were determined from three independent experiments. When different MIC endpoints were obtained among independent experiments, the modal MIC (the value observed most frequently) was reported.

**α-Glucosidase Inhibitory Assay**

The inhibitory activity of the leaf essential oil of *G. petelotii* against α-glucosidase from *Bacillus* (Sigma-Aldrich, G3651) was determined according to a previously established protocol ^[2]^. The assay was carried out in 96-well microplates. The reaction included 50 µL of the essential oil, 50 µL of α-glucosidase solution (0.5 U/mL) in phosphate buffer (0.1 M, pH 6.8). The reaction was incubated at 37 ^o^C for 15 minutes. Subsequently, 50 µL of 5 mM p-nitrophenyl-α-D-glucopyranoside (pNPG) was added as the substrate, and the reaction was further incubated at 37 ^o^C for 5 minutes. The reaction was then terminated by the addition of 50 µl of 0.2 M Na_2_CO_3_. Absorbance was recorded at 405 nm using a microplate reader (BioTek Instruments, USA). Acarbose was employed as the positive control. The percentage inhibition of α-glucosidase activity was calculated using the formula:

Inhibition (%) = (1 – A/Ac) × 100

where A is the absorbance of the test sample and Ac is the absorbance of the control. The experiments were performed in triplicate and the data are expressed as the mean ± standard deviation.

**Xanthine Oxidase Inhibitory Assay**

The inhibitory activity against xanthine oxidase (XO) from bovine milk (Sigma-Aldrich, X1875) was evaluated by a microplate-based spectrophotometric assay with slight modifications from a reported method ^[3]^. The reaction containing 50 µL of the essential oil, 35 µL of 70 mM phosphate buffer (pH 7.5) and 30 µL of enzyme solution (0.01 U/ml) was pre-incubated at 25˚C for 15 min, followed by the addition of 60 µl of 150 µM xanthine. After incubation for 30 min at 25 °C, the reaction was quenched with 25 µL of HCl 1 N and absorbance was read at 290 nm using a microplate reader (BioTek Instruments, USA). Allopurinol was used as a positive control. The percentage of inhibition was calculated as:

Inhibition (%) = (1 – A/Ac) × 100

where A is the absorbance of the test sample and Ac is the absorbance of the control. The experiments were performed in triplicate and the data are expressed as the mean ± standard deviation.

**Acetylcholinesterase Inhibition Assay**

The inhibitory activity against acetylcholinesterase (AChE) from *Electrophorus electricus* (electric eel) (Sigma-Aldrich, C3389) was evaluated using a microplate-based spectrophotometric assay adapted from Ellman’s method with slight modifications as previous work ^[4]^. In brief, the reaction containing 100 µL of 3 mM 5,5-dithiobis-2-nitrobenzoate, 20 µL of the EO and 20 µL of AChE (0.2 U/mL) was pre-incubated at 25˚C for 15 min, and then initiated by the addition of 20 µl of 15 mM acetylthiocholine iodide. Following incubation at 25˚C for 20 min, the reaction was terminated by the addition of 20 µl of 4% SDS. Subsequently, the absorbance of the reaction was measured at 415 nm using a microplate reader (BioTek Instruments, USA). Galanthamine was used as a positive control. The percentage of inhibition was calculated as:

Inhibition (%) = (1 – A/Ac) × 100

where A is the absorbance of the test sample and Ac is the absorbance of the control. The experiments were performed in triplicate and the data are expressed as the mean ± standard deviation.

**Inhibition of nitric oxide production**

The inhibitory effect of the test compounds on nitric oxide (NO) production was evaluated in LPS-stimulated RAW264.7 macrophages using the Griess reaction ^[5]^. Cells were cultured in DMEM containing 10% FBS and 1% penicillin–streptomycin at 37 °C, 5% CO_2_. RAW264.7 cells (1 × 10^5^ cells/well) were seeded in 96-well plates and pretreated with test samples for 1 h, followed by stimulation with LPS (1 μg/mL) for 24 h. Culture supernatants were collected and mixed with an equal volume of Griess reagent. The mixture was maintained for 10 min at room temperature, and the absorbance was measured at 540 nm. Nitrite concentrations were calculated from a NaNO_2_ standard curve. Dexamethasone was used as a positive control. The experiments were performed in triplicate and the data are expressed as the mean ± standard deviation.

The proliferation of cells treated with samples was determined by MTT assay. After removing the culture supernatant for NO quantification, each well was added 90 µL of fresh culture medium and 10 µL of MTT solution (5 mg/mL). Following a 4 h incubation, the medium was removed, and the resulting formazan crystals were dissolved in 100 µL of 100% DMSO. Absorbance was recorded at 540 nm using a microplate reader (Biotek, Winooski, VT, USA). The percentage of cell viability was determined by the following formula:

Cell viability (%) = 100 × (OD sample – OD blank)/(OD negative control – OD blank)

Where OD sample is the absorbance of cells treated with the test sample in the presence of LPS, OD negative control is the absorbance of cells treated with LPS and the vehicle alone (without the test sample), and OD blank is the absorbance of wells containing culture medium without cells.

**Table S1.** Antibacterial activity of the *Glycosmis petelotii* essential oil

| **Bacterial strains** | **MIC (µg/mL)** | |
| --- | --- | --- |
|  | **Essential oil** | **Ciprofloxacin** |
| *Escherichia coli* ATCC25922 | 128 | 0.5 |
| *Salmonella enterica* ATCC13076 | 128 | 1 |
| *Pseudomonas aeruginosa* ATCC27853 | 256 | 2 |
| *Enterococcus faecalis* ATCC29212 | 128 | 4 |
| *Staphylococcus aureus* ATCC25923 | 256 | 1 |
| *Bacillus cereus* ATCC14579 | 128 | 2 |

**Table S2.** Biological activity of the *Glycosmis petelotii* essential oil

| *α*-Glucosidase inhibition | IC_50_ (µg/mL) |
| --- | --- |
| *Glycosmis petelotii* essential oil | 707.5 ± 22.6 |
| Acarbose | 117.7 ± 4.0 |
| Xanthine oxidase inhibition | **IC_50_ (µg/mL)** |
| *Glycosmis petelotii* essential oil | >100 |
| Allopurinol | 1.13 ± 0.06 |
| Acetylcholinesterase Inhibition | **IC_50_ (µg/mL)** |
| *Glycosmis petelotii* essential oil | >100 |
| Galantamine | 1.85 ± 0.12 |
| Inhibition of nitric oxide production | **IC_50_ (µg/mL)** |
| *Glycosmis petelotii* essential oil | >100* |
| Dexamethasone | 12.32 ± 1.37 |

**Cell viability of LPS-stimulated RAW 264.7 macrophages remained above 90% at the highest tested concentration of Glycosmis petelotii essential oil (100 µg/mL), indicating that the observed inhibition of nitric oxide production was not associated with cytotoxicity.*

**Table S3.** Comparison of the experimental and reference EI mass spectra of niranin

| ***m/z*** | **Reference** ^[6]^ | **Experimental** | **Agreement** |
| --- | --- | --- | --- |
| 42 | + | + | Matched |
| 61 | + | + | Matched |
| 75 | + | + | Matched |
| 90 | + | + | Matched |
| 105 | + | - | Minor difference |
| 118 | Base peak | Base peak | Excellent |
| 133 | + | + | Matched |
| 162 | + | + | Matched |
| 209 | Molecular ion | Molecular ion | Excellent |

(+): Detected; (-): Not detected

**Figure S1.** Experimental EI mass spectrum of the peak tentatively identified as niranin

**Figure S2.** Experimental EI mass spectrum of niranin from Adams (2017) ^[6]^

**Figure S3.** The TIC chromatogram of the n-alkane standard mixture

**Figure S4.** Superimposition of the crystallographic and redocked poses of maltose within the α-glucosidase active site. The crystallographic pose is shown in blue, and the redocked pose is shown in yellow. The heavy-atom RMSD between the two poses was 0.91 Å.

1.	Hoang, H.N.T., et al., *A new 14-membered cembrane-type diterpenoid and other constituents from the aerial parts of Jatropha integerrima Jacq. and their antibacterial activity.* Natural Product Research: p. 1-7.

2.	Nguyen, T.K., et al., *Xanthine oxidase, α-glucosidase and α-amylase inhibitory activities of the essential oil from Piper lolot: In vitro and in silico studies.* Heliyon, 2023. **9**(8): p. e19148.

3.	Tran, L.T.T., et al., *Virtual Screening and in Vitro Evaluation to Identify a Potential Xanthine Oxidase Inhibitor Isolated from Vietnamese Uvaria cordata.* Natural Product Communications, 2022. **17**(2): p. 1934578X221080339.

4.	Nguyen, T.K., et al., *Deep Learning Model to Identify Potential Acetylcholinesterase Inhibitors: A Case Study of Isolated Compounds From Pongamia pinnata (L.) Pierre.* Natural Product Communications, 2022. **17**(7): p. 1934578X221117310.

5.	Giang, P.M., et al., *Evaluation of the Antioxidant, Hepatoprotective, and Anti-Inflammatory Activities of Bisresorcinol Isolated from the Trunk of Heliciopsis Terminalis.* Pharmaceutical Chemistry Journal, 2019. **53**(7): p. 628-634.

6.	Adams, R.P., *Identification of essential oil components by gas chromatography/mass spectrometry. 5 online ED*. Gruver, TX 2017: USA: Texensis Publishing.
